# Hypotension and Environmental Noise: A Replication Study

**DOI:** 10.3390/ijerph110908661

**Published:** 2014-08-26

**Authors:** Peter Lercher, Ulrich Widmann, Jürg Thudium

**Affiliations:** 1 Division of Social Medicine, Medical University Innsbruck, Sonnenburgstrasse 16, A-6020 Innsbruck, Austria; 2 AUDI AG, I/ET, D-85045 Ingolstadt, Germany; E-Mail: Ulrich.Widmann@audi.de; 3 Oekoscience-Institute, CH-7000 Chur, Werkstrasse 2, Switzerland; E-Mail: Thudium@oekoscience.ch

**Keywords:** hypotension, sex, age, traffic noise, rail noise, BMI, weather sensitivity, effect modification

## Abstract

Up to now, traffic noise effect studies focused on hypertension as health outcome. Hypotension has not been considered as a potential health outcome although in experiments some people also responded to noise with decreases of blood pressure. Currently, the characteristics of these persons are not known and whether this down regulation of blood pressure is an experimental artifact, selection, or can also be observed in population studies is unanswered. In a cross-sectional replication study, we randomly sampled participants (age 20–75, N = 807) from circular areas (radius = 500 m) around 31 noise measurement sites from four noise exposure strata (35–44, 45–54, 55–64, >64 Leq, dBA). Repeated blood pressure measurements were available for a smaller sample (N = 570). Standardized information on socio-demographics, housing, life style and health was obtained by door to door visits including anthropometric measurements. Noise and air pollution exposure was assigned by GIS based on both calculation and measurements. Reported hypotension or hypotension medication past year was the main outcome studied. Exposure-effect relationships were modeled with multiple non-linear logistic regression techniques using separate noise estimations for total, highway and rail exposure. Reported hypotension was significantly associated with rail and total noise exposure and strongly modified by weather sensitivity. Reported hypotension medication showed associations of similar size with rail and total noise exposure without effect modification by weather sensitivity. The size of the associations in the smaller sample with BMI as additional covariate was similar. Other important cofactors (sex, age, BMI, health) and moderators (weather sensitivity, adjacent main roads and associated annoyance) need to be considered as indispensible part of the observed relationship. This study confirms a potential new noise effect pathway and discusses potential patho-physiological routes of actions.

## 1. Introduction

In general and clinical practice, subjects reporting to suffer from hypotension are typically labeled as persons with “constitutional”, “essential” or “orthostatic hypotension”. Both chronic or intermittent hypotension have been associated with a broad spectrum of symptoms including fatigue, dizziness, lightheadedness, palpitations, headache, cold limbs, low mood, depressive symptoms and reduced cognitive performance in clinical [1,2], large cross-sectional [3,4,5] and longitudinal studies [6,7,8,9,10]. Recent neurophysiologic studies demonstrated reduced cortical activity and a decrease in cerebral blood-flow already in subjects with mild hypotension [11]. These data using improved detection techniques challenge the conventional wisdom that cerebral-vascular auto-regulation prevents reduced cerebral perfusion even at lower systolic blood pressures [12] and provide a patho-physiological basis for some of the observed impairments. [13] Nevertheless, persons suffering from essential hypotension typically have a lower cardiovascular risk profile and also a better survival [14]. Due to the lower mortality experience, clinicians often tended to neglect people complaining about chronic hypotension and label it as a non-disease [15] despite the observed association with considerable morbidity at the community level seen in the later studies. Although the most affected are younger people with a preponderance of women, hypotension in the elderly does carry some health risks as hypotension (mostly orthostatic) has been found to be associated with cognitive decline, dementia, falls, stroke and increase in overall mortality in more recent studies [16,17,18,19,20,21,22,23].

While the effects of occupational and environmental noise on high blood pressure are well studied [24] and exposure response curves are available for road and air traffic noise [25,26], the potential relation with low blood pressure has not been addressed. There are two good reasons for this: first, the fact that hypertension obviously is of larger public health importance and secondly, the mechanism of action (stress response) is well understood. Nevertheless, the earlier experimental studies on the effects of noise on blood pressure revealed often inconsistent results and used high intensities (>75 dBA). While some studies found increases [27,28,29], others [30,31] reported no change or decreases [32,33,34,35]. The observed discrepancies were not deeply discussed in the literature. Later experimental studies also used loud or occupational noise (for a review see [36]). One exception is the study of Chang [37] which used environmental noise and found small increases in blood pressure. All analyses of those studies focused, however, on increases in blood pressure and therefore did not report the overall response information. This information would have been required to see the proportion of participants who did not respond with increases or showed no effect. Since most studies were small and designed to investigate mean effects of noise exposure on blood pressure, information about subgroups who may have responded differentially was rarely available. The subgroups studied were persons with mild hypertension or a family history of hypertension, where a higher sensitivity toward stressors was expected. These groups consistently responded with increases in blood pressure [28,38]. A differential effect of noise with age was found in normotensive male industrial workers [39]. While the younger group showed an increase in systolic BP the older group (aged 45 to 65) exhibited a decrease after adjustment for potential confounding factors. No investigation dealt with participants labeled with essential or orthostatic hypotension. Thus, firm conclusions for everyday sound exposure at environmental levels were rather limited in the majority of studies as the noise exposure was typically high (75 to 100 dBA), short (10 to 30 min), and the study participants were young and healthy adults. Eventually, the typical noise applied in these experiments (often white or meaningless noise) was mostly ecologically invalid.

An exception is the series of carefully conducted field experiments under naturalistic working conditions at the Environmental Agency in Berlin. Smaller experiments with higher (occupational) noise exposure showed already some decreases (4 to 13%) in subgroups [32,34]. Another German investigation used short-term traffic noise of 72 dBA [33] and found a larger proportion of blood pressure decreases (33% systolic and 23% diastolic). Of central interest for the rationale of our investigation was, however, the larger (N = 46) experiment in Berlin with ecologic valid traffic noise exposure (60 dBA vs 50 dBA) of longer duration, where the full distribution of noise effects on blood pressure was reported [35]. They found in subgroups maximum decreases in blood pressure readings of up to 12 mm systolic and 9 mmHg diastolic after 6.5 hours of exposure to traffic noise (60 dBA) [40] compared with a control day exposure of less than 50 dBA. Moreover, this investigation could show that, by accepting both increases and decreases of blood pressure as noise effects, the explained variance increased in noise sensitive persons from 29 to 53%. Furthermore, in a follow-up of persons (1 to 3 weeks) the observed response pattern (increases or decreases) remained stable with a few exceptions.

We took these pieces of evidence as a rationale to make a first evaluation whether hypotension (defined as reported diagnosis or by recorded blood pressure) may be associated with transportation noise in a large field survey. We found self-reported hypotension non-linearly associated with noise exposure in the presence of a strong sex×age effect modification [41]. However, no relation between noise and continuous blood pressure readings could be established at recommended cut-off points for hypotension.

Reproducibility is one of the cornerstones of science [42,43,44]. However, exact direct replication is often not feasible in field studies or not always helpful when replication is done in exactly the same way as in the original study [45]. The aim of this paper is to report a conceptual replication analysis applied to data from a community health survey in a neighboring alpine valley with improved sampling design and more detailed information on both the sound exposure sources and the hypotension experience.

## 2. Methods

### 2.1. Area, Study Design and Sampling

The study area covers a stretch of about 40 km in the Lower Inn valley (east of Innsbruck, Austria) and consists of densely populated small towns and villages with a mix of industrial, small business, touristic and agricultural activities. The cross-sectional study was conducted in the fall of 1998. Sampling was based from a noise map prepared for an environmental health impact assessment by GIS-stratification of noise exposure (35–44, 45–54, 55–64, >64 Leq, dBA). Sampling was conducted in a two step process and the selected persons were called four times before being replaced. People (aged 20–75 years) were sampled randomly (See Supplementary Material, Figure S1) from the circular areas around 31 noise measurement sites (radius = 500 m). Persons (N = 807) from 648 households agreed to participate (50.5%) in the survey. The sampling from these circular areas should increase the validity of the noise assignments by minimizing the known errors of sound propagation procedures for larger distances. Only persons with a permanent residence of more than one year were included in the study. Prior written consent was taken from the participants before the interview and the anthropometric measurements were made.

### 2.2. Sound Exposure Assessment

The primary noise sources were road (highway, main road) and rail traffic. During the past decade a slight increase in night time freight trains could be observed. At the same time, a night ban on non-noise-abated trucks led to a slight decrease (~3 dBA) in night time noise levels of highway traffic.

The final individual assignment of the source specific noise exposure (dBA, day and night, Ldn) was made after calibration of the modeling results against the measurements from the 31 sites in the center of the circular areas. The measurement points were selected from two experienced acousticians to cover the variety of topography (valley/slope), settlement structure (housing types, rural/suburban/town) and population density of the area of investigation. The measurements were carried out in the year preceding our survey and covered day, evening and night. The full measurement period was accompanied with traffic counts for all sources and types of traffic. These traffic and measurement data were then used as calibration input against the original noise map which was based on yearly average daily traffic of the respective sources. All procedures were carried out according to Austrian guidelines (ÖAL Nr. 28 + 30, ÖNORM S 5011) with a resolution of 25 m × 25 m.

In the present analysis, we used the calibrated sound level of the total, the highway and the railway exposure (Ldn) and additionally entered distance to the main road as potential moderator into the model, since it was impossible to separate the true contribution of this road from the other sources with sufficient certainty. In order to account also for smaller road traffic sources (“local roads”), we kept also the general annoyance question directed towards these nearby sources as interaction term in the model.

### 2.3. Air Pollution Exposure Assessment

Research in Austria, France, Italy and Switzerland has shown that due to the specific meteorological, climatic and topographic conditions in alpine valleys, the same amount of emission generates up to four times higher air concentrations at the receiver point than in flat land [46,47]. The ratio between air concentration and emission varies over a large range and only a part of the observed variations in the air and noise pollution at the two sites can be explained by the varying emission at the motorway [48].

Exposure was assessed by a Swiss expert group (OEKOSCIENCE AG, Zürich, Switzerland), who had long-term experience in monitoring and calibrating air pollution exposure in the alpine areas with special consideration of meteorological and topographical conditions [49]. An adapted Gaussian propagation model procedure was used under the prevailing meteorological conditions (three seasons) for the respective area. The results were assigned via GIS to the addresses of the study participants. The calculations were done for a resolution of 100 m × 100 m. Beyond a distance of 1 km of the line source of the motorway the prediction is slightly worse, but concentrations are also smaller. The calibration was based on nearby fixed monitoring stations. Later modeling (“Tau”-model) was based on empirical proportions between air concentrations and emissions caused by the specific sources [47,50].

### 2.4. Main Health Outcome Measures

Illnesses were determined using an exhaustive list (e.g., ”low blood pressure”, “high blood pressure”) which was preceded by the general question: “Has a doctor diagnosed one of the following health problems” and linked to three answer boxes: “during the past 12 months”; “ever”, “never”. 

Information on medication was questioned in the same way “During the PAST 12 MONTHS, have you taken medication because of the following health problems” with an exhaustive list including the two options: “against low blood pressure” and “against high blood pressure”. 

Body mass and blood pressure was measured after a standard protocol by trained interviewers in the home of the subject. Only 572 persons participated. Blood pressure was obtained on the right arm in a sitting position prior to and after the interview—after three minutes rest. Within 5 to 10 days after the interview a third and fourth reading was obtained. This additional requirement for successive readings later in time reduced our sample to 572 persons with complete anthropometric records. A calibrated mercury sphygmomanometers (Sysditon, Fa. F. Bosch, Jungingen, Germany) with a large scale were used. The measurements were based on the first and fifth Korotkoff phase using a fixed deflation rate of 3 mm Hg/s. Reporting was required to 1 mmHg to avoid digit preference. A uniform cuff-size (12 cm × 28 cm) was used and no corrections were applied due to larger arm circumferences.

### 2.5. Confounding and Moderation

The extensive standardized questionnaire covered socio-demographic data, housing, satisfaction with the environment, general noise annoyance, interference of activities, coping with noise, occupational exposures, lifestyle, general dispositions such as noise and weather sensitivity and health status. Education was measured with five grades (basic, skilled labor, vocational school, A-level, University degree). The last two grades were combined in the category “higher education.” Density is calculated as people/room. General sensitivity towards noise, air pollution, vibrations and weather changes were assessed with a visual analogue 11 point intensity scale (not at all = 0 to 10 = extremely). Health status was judged on a standard 5-grade scale. Family history of hypertension: “Did your father or your mother have … high blood pressure? (Mother-father-both-do not know)”. In addition, physical and mental health was assessed by two subscales (14 items) of the 28 item version of the General Health Questionnaire (GHQ), namely the somatic and anxiety scales. Items were graded in four steps. For this analysis we also used a full scale of all 14 items (Cronbach’s alpha = 0.89). Sleep quality was measured with a summary scale (Cronbach’s alpha = 0.86) derived from five sleep frequency items: “problems in getting to sleep”, “waking up”, “problems in getting back to sleep”, “waking up too early”, “tiredness/fatigue in the morning”. The verbal frequency options were: “nearly every day”, “several times a week”, “several times a month”, “less often than that”, “never”. As we observed area related differences with hypotension reporting in the earlier study, we created an area variable based on geographical features.

### 2.6. Statistical Analysis

Exposure and survey data were linked through a Geographical Information System and statistical analysis was conducted with R-Software [51]. Dichotomous variables for Table 1 were examined by the Pearson Chi-square test. For numeric type data medians and inter-quartile ranges are presented and the p-values of the Wilcoxon Rank Sum test are reported.

In the present exposure response analyses we used the sound level of all sources (overall or total Ldn). In addition to the previous study, we were able to test the differential contribution of the main contributing traffic sources as well (road versus rail traffic). Road traffic predominantly included highway traffic—but as in some areas a relevant noise exposure came also from other roads—distance to main road and annoyance due to other close-by local roads were included in all models. Likewise, we kept these additional road variables in the rail models. 

Exposure-effect relationships were modeled with multiple logistic regression techniques using Harrell’s RMS-library [52]. To account for non-linearity in selected predictors splines were applied. Approximate 95% confidence intervals were estimated using smoothing spline routines with three knots and the exposure-effect plots were generated with the RMS-library. Predicted probabilities are derived from the estimated odds with a specific function in the RMS-library (plogis). The predicted probabilities in the exposure-effect plots of self-reported hypotension or hypertension medication are adjusted to the median (continuous variables) or the reference category (non-continuous variables) of the other variables in the model.

Model building was based on the previous analysis and other prior substantive knowledge with a standard model including the outcome, one exposure indicator, distance to the main road, age, sex, education, body mass index (BMI), family history of hypertension, hypertension treatment, health status or general health questionnaire score (GHQ), sleep score and special sensitivities (noise, weather). Since differences in hypotension prevalence or medication prescriptions were noted in the communities, a variable indicating different study areas (east-middle-west) was included in the baseline model.

**Table 1:** Description of the relationships between main study variables and the two health outcomes (full sample).

Categorical variables	Reported	Reported	Test Statistic	Hypotension	Hypotension	Chi-Square-
	Hypotension: No	Hypotension: Yes	Chi-Square-Statistic	Medication: No	Medication: Yes	Statistic
	n (%)	n (%)	*p* value	n (%)	n (%)	*p* value
Total	693 (86)	114 (14)		724 (90)	79 (10)	
Gender			<0.001			<0.001
Female	336 (48.5)	93 (81.6)		366 (50.6)	62 (78.5)	
Male	357 (51.5)	21 (18.4)		358 (49.4)	17 (21.5)	
Health status			<0.001			< 0.001
very good/good	395 (57)	40 (35.4)		417 (57.6)	18 (23.1)	
less than good	298 (43)	73 (64.6)		307 (42.4)	60 (76.9)	
Educational level			0.652			0.537
Basic	174 (25.4)	26 (22.8)		179 (24.9)	21 (26.9)	
Skilled labour	227 (33.1)	41 (36)		236 (32.9)	29 (37.2)	
Vocational	152 (22.2)	29 (25.4)		163 (22.7)	18 (23.1)	
A-level	133 (19.4)	18 (15.8)		140 (19.5)	10 (12.8)	
Family history of hypertension			0.033			0.880
Yes	223 (32.3)	49 (43)		204 (28.2)	24 (30.4)	
No	468 (67.7)	65 (57)		313 (43.3)	32 (40.5)	
Area of valley			0.935			
East	197 (28.5)	31 (27.2)		204 (28.2)	24 (30.4)	0.615
bottom	299 (43.2)	49 (43)		240 (33.2)	29 (36.7)	
West	196 (28.3)	34 (29.8)		483 (66.8)	50 (63.3)	
Antihypertensive treatment			<0.001			0.005
No	567 (82.1)	112 (99.1)		604 (83.4)	75 (96.2)	
Yes	124 (17.9)	1 (0.9)		120 (16.6)	3 (3.8)	
**Continuous variables**	**Reported**	**Reported**	**Ranksum Test *p* Value**	**Hypotension**	**Hypotension**	**Ranksum Test *p* Value**
	**Hypotension: No**	**Hypotension: Yes**		**Medication: No**	**Medication: Yes**	
	**Median (IQR)**	**Median (IQR)**		**Median (IQR)**	**Median (IQR)**	
Age			0.109			0.050
median (IQR)	44 (34, 57.5)	40 (33, 53.8)		43 (34, 57)	50 (37.5, 59.5)	
Total sound level: dBA, Ldn+			0.183			0.870
median (IQR)	57.7 (54.5, 61.3)	58.3 (54.9, 62.6)		57.7 (54.6, 61.4)	57.3 (54.1, 61.8)	
Rail sound level: dBA, Ldn+			0.227			0.887
median (IQR)	54.3 (51.7, 58.7)	54.5 (52.1, 61.5)		54.4 (51.7, 59)	53.6 (51.7, 60.2)	
Highway sound level: dBA, Ldn+			0.653			0.786
median (IQR)	53.8 (50, 56.3)	54 (49.9, 56.7)		53.8 (50, 56.3)	53.9 (49.3, 55.7)	
Distance to main road			0.024			0.484
median (IQR)	435.7 (197.1, 1172.7)	306.1 (162.6, 1132.2)		428.5 (185.1, 1172.7)	380 (178.4, 1140)	
Annoyance by local road			0.118			0.012
median (IQR)	3 (1, 6)	4 (2, 6)		3 (1, 6)	5 (2, 7)	
NO_2_: annual average, µg/m³			0.343			0.698
median (IQR)	33.9 (32, 35.7)	34.1 (32.4, 36.2)		33.9 (32.1, 35.8)	33.8 (31.9, 35.7)	
Noise sensitivity			0.006			0.006
median (IQR)	5 (2, 8)	6 (3, 8)		5 (2, 8)	6 (3, 9)	
Weather sensitivity			<0.001			<0.001
median (IQR)	3 (1, 5)	5 (3, 8)		3 (1, 5)	6 (3, 8)	
GHQ score *			<0.001			<0.001
median (IQR)	21 (18, 26)	24.5 (20.8, 31)		21 (18, 26)	26 (21, 32)	
Sleep score *			<0.001			<0.001
median (IQR)	6 (3, 10)	9 (4, 13)		6 (3, 10)	10 (5, 14)	

* The higher the worse; + Ldn: day-night adjusted sound level in decibel.

Eventually, the potential role of air pollution was evaluated by yearly mean NO_2_ concentration. Based on the previous analyses only selected interaction terms (age × sensitivity, noise × sensitivity, age × sex, age × sensitivity) were included *a priori*. In addition health × sensitivity and age × noise were tested. In response to a reviewer’s comments, distance to the main road and annoyance by local roads were entered as additional interactions with the respective sound level under analysis. Interactions (IA) were tested one by one and kept in the model when either indicators of fit improved or the adjusted R^2^ increased-balancing variance inflation. The statistical criterion for the inclusion of IA in the model was relaxed (to *p* = 0.2) since departure from additivity is considered of relevance for accurate prediction in a public-health context when involved exposures and outcomes are sufficiently prevalent [53,54]. Sensitivity analyses were carried out after [55] and [56]. Specifically, the final models were validated by bootstrapping to check for over-fitting and evaluated against multiple discrimination criteria (AIC, BIC, R^2^, model χ², Somers’ Dxy, Spearman’s ρ, Gamma, Tau-a, C (area under ROC curve) and VIF. The C-index as indicator of accuracy remained high (0.89) and AIC and BIC still improved when all five interactions were entered in the model with reported hypotension—although variance inflation went up. Based on these discrimination and accuracy criteria and principal component analyses (using R-psy) in the early process of model building the simple health question was kept in the model instead of the overall GHQ-score or the two subscales (somatic symptoms, anxiety/sleep). Also several single GHQ items (anxiety, ill health, irritable, stressed) did not perform better than the five graded standard health question.

## 3. Results

### 3.1. Sample Characteristics by Health Outcome

Univariate socio-demographic, health and exposure characteristics of the full sample are described in relation to the investigated health outcomes in Table 1. Concordantly, with both outcomes, significant relations were observed with sex, health status or GHQ-score, anti-hypertensive treatment, noise or weather sensitivity and sleep score. Discordant relations were observed with distance to main road and family history of hypertension. Age was significantly higher (*p* = 0.05) in the medication group while the group with reported hypotension was younger (*p* = 0.11). Annoyance by local road was higher in both outcomes—but significantly in the medication group only. No significant univariate relations were observed with educational level, area, overall noise level and air pollution (NO_2_).

In the subsample (N = 570 with anthropometric measurements), the relations were nearly identical. In addition BMI was highly significant in both outcome groups (see Supplementary Material, Table S1) and included in the model. BMI was an important variable also in the earlier analysis [41].

### 3.2. Hypotension Past Year: Full Sample (N = 748)

Table 2 provides the final model parameters for overall, rail and road noise exposure. Sex and weather sensitivity make the dominant contribution in all models before hypertension treatment (inverse relation) and health status. The adjusted pseudo R^2^ of all models is quite high compared with other noise and health studies.

**Table 2:** Multiple logistic regression results for total, rail and highway sound exposure models with reported hypotension, including twelve covariates and five interaction terms (N = 748).

Factor	Total Sound Exposure			Railway Sound Exposure			Highway Sound Exposure		
	Chi-Square	d.f.	*p* Value	Chi-Square	d.f.	*p* Value	Chi-Square	d.f.	*p* Value
Sound level as Ldn	24.25	8	0.0021	23.88	8	0.0024	10.36	8	0.2407
Nonlinear component sound level	16.24	4	0.0027	14.02	4	0.0072	4.26	4	0.3717
Distance to main road	10.32	3	0.0160	11.36	3	0.0099	7.15	3	0.0671
Annoyance by local roads	8.25	3	0.0412	7.72	3	0.0521	2.48	3	0.4786
Sex	32.33	2	<0.0001	31.85	2	<0.0001	30.18	2	<0.0001
Age	14.82	2	0.0006	14.30	2	0.0008	12.73	2	0.0017
Educational level	4.08	3	0.2532	4.25	3	0.2354	3.87	3	0.2754
Family history of hypertension	4.96	1	0.0259	4.83	1	0.0279	3.48	1	0.0620
Region (west-bottom-east)	4.19	2	0.1230	4.29	2	0.1171	1.44	2	0.4862
Antihypertensive treatment	15.74	1	0.0001	14.67	1	0.0001	14.31	1	0.0002
Weather sensitivity	29.84	4	<0.0001	28.18	4	<0.0001	24.56	4	0.0001
Health status	12.99	2	0.0015	13.57	2	0.0011	11.93	2	0.0026
Sleep score	2.95	1	0.0858	2.64	1	0.1044	4.56	1	0.0328
Age × sex	14.52	1	0.0001	14.02	1	0.0002	12.20	1	0.0005
Weather sensitivity × health	6.89	1	0.0087	7.25	1	0.0071	6.41	1	0.0114
Sound level × weather sensitivity	7.93	2	0.0190	5.76	2	0.0561	4.32	2	0.1153
Nonlinear Interaction	6.60	1	0.0102	3.73	1	0.0533	4.22	1	0.0400
Sound level × distance main road	8.18	2	0.0167	8.99	2	0.0112	5.46	2	0.0651
Nonlinear Interaction	7.81	1	0.0052	8.71	1	0.0032	0.00	1	0.9447
Sound level × annoyance local roads	6.94	2	0.0312	6.07	2	0.0480	0.99	2	0.6091
Nonlinear Interaction	2.94	1	0.0863	0.64	1	0.4244	0.70	1	0.4016
TOTAL nonlinear	16.24	4	0.0027	14.02	4	0.0072	4.26	4	0.3717
TOTAL interaction	32.99	8	0.0001	32.74	8	0.0001	25.17	8	0.0015
TOTAL nonlinear+interaction	35.22	9	0.0001	34.53	9	0.0001	25.74	9	0.0022
TOTAL	95.11	24	<0.0001	96.39	24	<0.0001	94.59	24	<0.0001

Model pseudo R^2^ = 0.39; Model pseudo R^2^ = 0.39; Model pseudo R^2^ = 0.36.

Both total and rail noise are significant contributors in the full sample (Table 2). Both exposures show a significant non-linear component and a relevant moderation with weather sensitivity in Public health terms. A statistically significant interaction is also observed between health and weather sensitivity. The most important moderation in all models is with age and sex. Reported hypotension prevalence is highest among younger women and increases with age in men (see Supplementary Material Figure S2).

Highway noise is unrelated to hypotension (Table 2 and Table 5). However, there is indication for noise from local/main roads to contribute beyond the other sources as indicated by the significant moderation of distance and reported annoyance through these sources in both the total and the rail model. No moderation was found, however, with exposure to noise from the highway.

**Figure 1:**
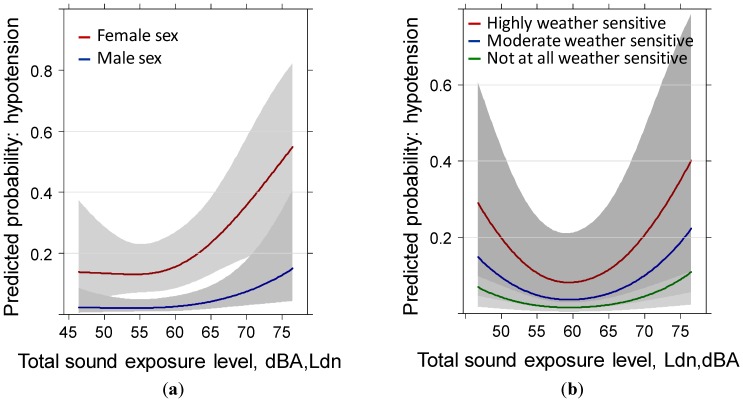
Predicted probability of reported hypotension past year with total sound level exposure by (**a**) sex and (**b**) weather sensitivity. Models are adjusted for age, education, anti-hypertensive treatment, family history of hypertension, health status, sleep score, distance to main road, annoyance by local roads, region and the IA-terms age × sex, weather sensitivity × health, weather sensitivity × sound exposure, distance × sound exposure and annoyance × sound exposure.

Due to the significant interaction of the sound level with weather sensitivity and annoyance we cannot attribute the effect observed to the sound level alone. Another significant interaction between weather sensitivity and health status is observed and indicates that both health status and weather sensitivity are critical contributors in this model. Figure 1 show the overall exposure response information by sex and weather sensitivity. The interaction between sound level and weather sensitivity is statistically significant.

**Figure 2:**
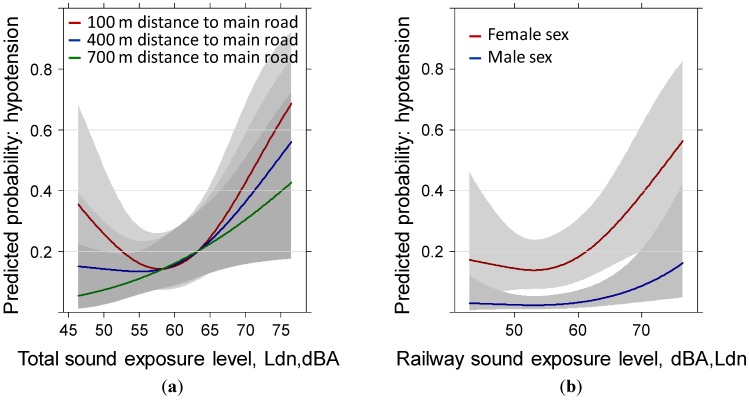
Predicted probability of reported hypotension past year with total sound exposure moderated by: (**a**) distance to the main road and (**b**) railway sound exposure by sex. Models are adjusted for age, education, anti-hypertensive treatment, family history of hypertension, weather sensitivity, health status, sleep score, distance to main road, annoyance by local roads, region and the IA-terms age × sex, weather sensitivity × health, weather sensitivity × sound exposure, distance × sound exposure, and annoyance × sound exposure.

Another significant moderation is evident from Figure 2a. The closer an additional main road is to the home of the participants the stronger is the moderating effect, especially at lower and higher sound levels. The exposure-response for the rail—hypotension relationship mimics exactly the one with the total sound exposure (Figure 2b).

A comparison of the 10 dBA increase for all sound sources between 55 and 75 dBA is provided in the summary Table 5. Due to the non-linearity and the interaction involved, the plain increase in the odds ratio for the sound exposure of the adjusted model should be taken only as a conservative approximation.

### 3.3. Hypotension Past Year: Reduced Sample (N = 528) Including Body Mass Index

The results are similar compared with the full sample. Note: the model applied to the reduced sample contains BMI as additional variable (Table 3).

Sex and weather sensitivity remain the most important cofactors in terms of the Wald chi-square statistic. The importance of total and railway sound level (highway still insignificant) increased. The contribution of health status decreased—with the inclusion of BMI in this model. Overall, the adjusted pseudo R^2^ increased slightly to 0.42. Distance to main roads is significant in the rail (*p* = 0.038) and nearly significant in the total sound model (*p* = 0.054).

The sound level × weather sensitivity interaction retained its importance in Public health terms. In addition, the moderation by distance to the main road and the annoyance experienced by local roads remain statistically significant.

**Table 3:** Multiple logistic regression results for total, rail and highway sound exposure models with reported hypotension, including thirteen covariates and five interaction terms (N = 528).

Factor	Total Sound Exposure			Railway Sound Exposure			Highway Sound Exposure		
	Chi-Square	d.f.	*p* Value	Chi-Square	d.f.	*p* Value	Chi-Square	d.f.	*p* Value
Sound level as Ldn	24.00	8	0.0023	23.53	8	0.0027	9.86	8	0.2750
Nonlinear component sound level	14.72	4	0.0053	12.57	4	0.0136	2.85	4	0.5837
Distance to main road	8.01	3	0.0458	8.68	3	0.0338	6.97	3	0.0729
Annoyance by local roads	8.17	3	0.0427	7.61	3	0.0547	2.18	3	0.5355
Sex	18.68	2	0.0001	18.22	2	0.0001	16.65	2	0.0002
Age	5.95	2	0.0510	5.06	2	0.0795	3.86	2	0.1448
Educational level	5.88	3	0.1178	6.28	3	0.0987	4.43	3	0.2183
Family history of hypertension	5.14	1	0.0234	4.98	1	0.0256	3.39	1	0.0656
Region (west-bottom-east)	3.44	2	0.1788	3.75	2	0.1536	0.45	2	0.7994
Weather sensitivity	21.66	4	0.0002	20.46	4	0.0004	17.31	4	0.0017
Health status	5.75	2	0.0564	5.77	2	0.0557	5.54	2	0.0625
Sleep score	1.44	1	0.2308	1.30	1	0.2541	2.55	1	0.1101
BMI	5.20	1	0.0226	5.35	1	0.0208	5.62	1	0.0177
Antihypertensive treatment	12.90	1	0.0003	11.60	1	0.0007	10.80	1	0.0010
Age × sex	5.93	1	0.0149	5.05	1	0.0246	3.86	1	0.0494
Weather sensitivity × health	3.61	1	0.0575	3.62	1	0.0570	3.78	1	0.0519
Sound level × weather sensitivity	7.13	2	0.0283	5.90	2	0.0522	2.75	2	0.2532
Nonlinear Interaction	4.83	1	0.0279	2.92	1	0.0873	2.73	1	0.0986
Sound level × distance main road	5.88	2	0.0528	6.23	2	0.0444	5.54	2	0.0628
Nonlinear Interaction	5.70	1	0.0170	6.08	1	0.0137	0.00	1	0.9821
Sound level × annoyance local roads	7.01	2	0.0300	6.36	2	0.0415	0.62	2	0.7348
Nonlinear Interaction	3.42	1	0.0643	0.87	1	0.3505	0.61	1	0.4343
TOTAL nonlinear	14.72	4	0.0053	12.57	4	0.0136	2.85	4	0.5837
TOTAL interaction	20.49	8	0.0086	20.07	8	0.0101	13.82	8	0.0867
TOTAL nonlinear+interaction	23.85	9	0.0045	22.98	9	0.0062	15.25	9	0.0842
TOTAL	69.02	25	<0.0001	69.62	25	<0.0001	69.56	25	<0.0001

Model pseudo R^2^ = 0.42; Model pseudo R^2^ = 0.42; Model pseudo R^2^ = 0.37.

**Figure 3:**
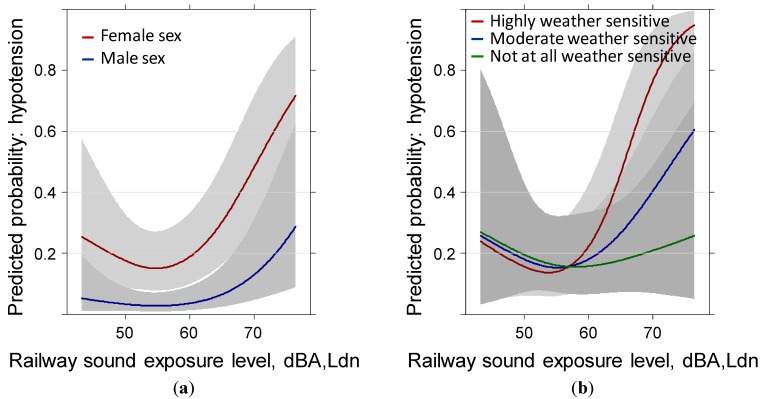
Predicted probability of reported hypotension past year with rail sound level exposure by: (**a**) sex and (**b**) weather sensitivity. Models are adjusted for age, education, BMI, anti-hypertensive treatment, family history of hypertension, health status, sleep score, distance to main road, annoyance by local roads, region and the IA-terms age × sex, weather sensitivity × health, weather sensitivity × sound exposure, distance × sound exposure, annoyance × sound exposure.

Visual inspection of the rail exposure response curves (Figure 3) shows steeper slopes in both curves and the interaction between sound and weather sensitivity looks more impressive in spite of similar parameter importance in the model. The smaller sample increased the variance particularly for the interaction terms. You can see similar variance inflation with the odds ratio increase for 10 dBA of the sound sources between 55 and 75 dBA in the summary Table 5.

It should be mentioned here that an identical model with “hypotension ever” as health outcome revealed similar qualitative results concerning the exposure response relation without reaching statistical significance (not shown). Nonetheless, the importance of the major covariates (sex, weather sensitivity, age, BMI) was preserved including the interactions between age and sex and between sound source and weather sensitivity and distance to main road. The pseudo R^2^ of the model was, however, moderately reduced (0.30). This is to be expected due to the larger exposure misclassification for the earlier residential exposure estimation (e.g., after moves) compared with the actual measured and calculated sound exposure at the current address. 

### 3.4. Hypotension Medication Past Year: Reduced Sample (N = 528) Including Body Mass Index

In order to avoid reporting too many similar findings, we only present the model results from the smaller sample containing BMI with the larger model pseudo R^2^ (Table 4). The medication model differs in some important aspects from the model with reported hypotension as outcome: Apart from the lower reported prevalence of medications (~70%) compared with reported hypotension, age became a more important cofactor than sex and the age × sex interaction lost almost completely its importance. The higher importance of age originates from the fact that general practitioners were more likely to give medications to patients complaining about symptoms related to low blood pressure with increasing age. All other interactions could not be replicated in these models although the cofactors weather sensitivity, health status, antihypertensive treatment and BMI remained highly important main predictors of medication use. 

**Figure 4:**
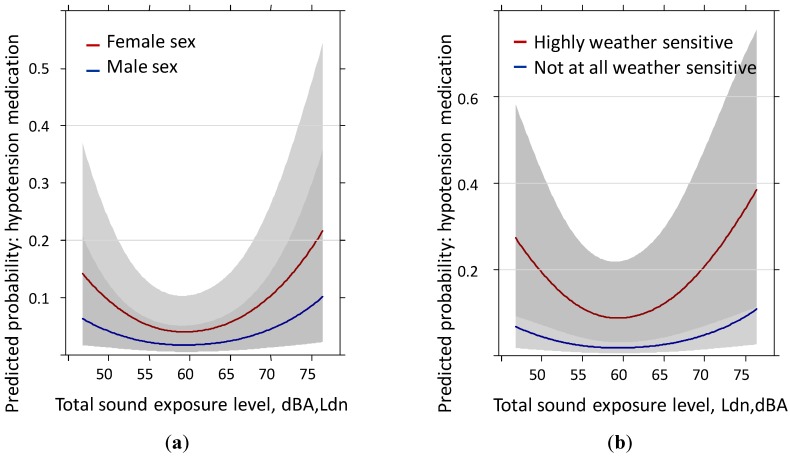
Predicted probability of hypotension medication past year with total sound level exposure by: (**a**) sex and (**b**) weather sensitivity. Models are adjusted for age, education, BMI, anti-hypertensive treatment, family history of hypertension, health status, sleep score, distance to main road, annoyance by local roads, region and the IA-term age × sex.

Distance to the main road and annoyance by local roads were completely unrelated to medication prescription. Nonetheless, the total sound level and the railway sound continued to be significant predictors and the highway exposure nearly approached significance (note: inverse relation). Furthermore, the shape of the exposure response curve changed and a non-significant but noticeable non-linear increase on the lower side of the sound exposure levels is visible (Figure 4). While this increase on the lower sound exposure side was already to some extent visible in the models with reported hypotension and nicely explained by the distance to main road×sound source interaction, there is no substantive explanation for this behavior in the medication model.

Detailed area analyses also failed to reveal a plausible explanation for this nonlinear increase on the lower end of the exposure levels. We assume selection factors related to medication prescription are involved. Medical doctors more likely treated persons who were older (See Supplementary Material Figure S3), in poorer health or that scored high on weather sensitivity. Moreover, large variations in treatment proportions (33 to 100%) could be observed at the community level. The odds ratio increase for 10 dBA of total and rail sound level between 55 and 75 dBA is provided for both samples in Table 5.

**Table 4:** Multiple logistic regression results for total, rail and highway sound exposure models with hypotension medication, including twelve covariates (N = 528).

Factor	Total Sound Exposure			Railway Sound Exposure			Highway Sound Exposure		
	Wald Chi-Square	d.f.	*p* Value	Wald Chi-Square	d.f.	*p* Value	Wald Chi-Square	d.f.	*p* Value
Sound level as Ldn	10.49	2	0.0053	7.97	2	0.0186	5.09	2	0.0786
Nonlinear component sound level	10.11	1	0.0015	6.92	1	0.0085	3.93	1	0.0474
Distance to main road	0.00	1	0.9733	0.02	1	0.8912	0.25	1	0.6177
Annoyance by local roads	0.62	1	0.4314	0.55	1	0.4599	0.46	1	0.4956
Sex	6.43	2	0.0402	6.42	2	0.0403	7.02	2	0.0299
Age	10.58	2	0.0050	10.23	2	0.0060	9.22	2	0.0099
Educational Level	5.91	3	0.1160	5.74	3	0.1252	6.11	3	0.1063
Family History of Hypertension	0.06	1	0.8142	0.07	1	0.7918	0.02	1	0.8789
Region (west-bottom-east)	0.30	2	0.8610	0.16	2	0.9216	0.86	2	0.6499
BMI	7.15	1	0.0075	7.53	1	0.0061	6.64	1	0.0100
Antihypertensive Treatment	12.57	1	0.0004	12.45	1	0.0004	12.78	1	0.0004
Weather Sensitivity	9.89	1	0.0017	9.75	1	0.0018	9.41	1	0.0022
Health status	8.86	1	0.0029	9.18	1	0.0024	10.28	1	0.0013
Sleep Score	1.68	1	0.1945	1.49	1	0.2228	2.58	1	0.1080
Age × sex	0.45	1	0.5026	0.49	1	0.4855	0.19	1	0.6631
TOTAL nonlinear + interaction	10.42	2	0.0055	7.28	2	0.0262	4.25	2	0.1196
TOTAL	65.09	18	<0.0001	64.52	18	<0.0001	62.56	18	<0.0001

BMI = body mass index; Model pseudo R^2^ = 0.39; Model pseudo R^2^ = 0.38; Model pseudo R^2^ = 0.37.

**Table 5:** A summary of the increase in the odds ratio (95% CI) at different sound levels in all adjusted models.

Sound Source and Health Outcome	Increase in Odds Ratio (95% CI) at Different Sound Levels		
	55–65 Ldn, dBA	60–70 Ldn, dBA	65–75 Ldn, dBA
**Full sample**			
Total sound: reported hypotension	2.01 (1.23–3.30)	3.00 (1.42–6.32)	3.31 (1.40–7.84)
Railway sound: reported hypotension	2.22 (1.36–3.62)	2.84 (1.43–5.67)	2.98 (1.43–6.24)
Highway sound: reported hypotension	0.63 (0.15–2.60)	0.61 (0.14–2.73)	0.61 (0.14–2.73)
Total sound: hypotension medication	1.08 (0.73–1.59)	2.00 (1.09–3.70)	2.35 (1.16–4.74)
Railway sound: hypotension medication	1.43 (0.95–2.15)	1.95 (1.09–3.51)	2.08 (1.11–3.88)
Highway sound: hypotension medication	1.63 (0.49–5.44)	1.72 (0.48–6.10)	1.72 (0.48–6.10)
**Reduced sample (incl. BMI)**			
Total sound: reported hypotension	2.05 (1.26–3.34)	4.28 (2.04–9.01)	5.57 (2.22–13.94)
Railway sound: reported hypotension	2.44 (1.53–3.89)	3.99 (1.96–8.13)	4.64 (2.06–10.44)
Highway sound: reported hypotension	1.13 (0.30–4.24)	1.13 (0.28–4.57)	1.13 (0.28–4.57)
Total sound: hypotension medication	1.11 (0.71–1.74)	2.74 (1.36–5.52)	3.79 (1.60–86)
Railway sound: hypotension medication	1.59 (1.01–2.49)	2.58 (1.30–5.11)	2.99 (1.38–6.50)
Highway sound: hypotension medication	2.57 (0.66–10.00)	2.80 (0.66–11.80)	2.80 (0.66–11.80)

## 4. Discussion

Our aim was to subject the novel results of a relationship between transportation noise exposure and hypotension to a retest in a smaller intensive survey conducted nine years later in another alpine valley with the same approach and approximately the same core variables.

We could replicate the central result of a statistically significant non-linear relationship of overall noise exposure with self-reported hypotension in the presence of some effect modifications. In addition, the distance to the main road turned out to be a relevant moderator. Therefore, the observed effect cannot be attributed to the sound levels alone but must be interpreted in the context of the other interactions involved.

In the hypotension medication models also a direct non-linear relationship with noise was observed without an interaction involved. In the total sound models the exposure-effect association starts, however, to level off significantly (see Table 5) at slightly higher sound levels compared to the previous study (60 to 70 dBA vs 55 to 65 dBA) while the rail models exhibit significant associations already in the 55 to 65 dBA range. Therefore, these results extend the earlier reported ones and direct further attention to the significant rail sound exposure. While we did not observe any significant association with the highway sound exposure at all, there was a significant moderation by the distance of the home from the main road.

Thus, why is railway noise contributing stronger to the overall noise effects on hypotension while the highway noise was unrelated? Rail passages can be characterized as intermittent noise with high peak levels. It dominates strongly during night and shows also higher peak levels (see supplemental Table S2). The peak levels of the trains during night are 13 dBA higher than those from the highway. The intermittent sound characteristic is more disturbing, especially at night [57,58]. Other studies have observed stronger effects on cardio-vascular indices, autonomic responses and on the sleep structure of railway noise than from the more continuous sound of larger roads [13,59,60,61], especially when vibrations are involved [62,63]. Furthermore, the longer pass-by time of freight trains is another factor during the night deserving attention. Support for this stronger effect of long freight trains comes from a larger study in the same area where we found sleep medication intake only related to rail noise but not to highway noise [64].

Notably in this context, closer distance of the home to the main road was a significant moderator in both the total and the rail sound models. The sound exposure characteristic of main roads can also be described as intermittent. Main road sound passing villages can be perceived as more annoying due to its acceleration and deceleration sounds, especially in more scattered residential living in rural and suburban areas [65,66]. Support for the potential adverse health effects of main roads comes from a recent finding of a stronger slope of the sound exposure annoyance curve for main roads in spite of lower sound levels compared with railway noise [67].

Concerning the importance of other covariates in the models, sex and weather sensitivity showed up again as most important predictors. Weather sensitivity gained this importance mainly through its significant interaction with health status and with rail and total sound level in both the full and the reduced sample. Why might (female) sex and weather sensitivity contribute so strongly to the relation between environmental noise exposure and hypotension? Women and weather sensitive persons are known to exhibit reduced autonomic nervous system regulation capacity.

A gender difference in autonomic functions related to blood pressure regulation is well documented [68,69,70,71]. Women have a more active parasympathetic system which let them compensate orthostatic hypotension less effectively [72]. In combination with a lower body mass (see later discussion) the autonomous regulation may be even more compromised.

Weather sensitivity has been used as general indicator of vegetative instability in European practical medicine [56] and standardized questionnaires (FBL of Fahrenberg, [73]) have been developed. Although, the direct relationship between sensitivity to weather and hypotension is not well-studied, it has been shown that under weather changes (e.g., hot weather) the associated symptoms of hypotension tend to worsen [74,75]. Furthermore, subjects reporting sensitivity to weather showed the strongest relation (OR = 7.12 (4.81–10.53)) with “circulatory problems” out of a list of 17 reported co-morbidities [76]. In a summary of research on cardiovascular effects of environmental noise in Austria we have shown earlier that weather sensitivity is often a stronger predictor with cardiovascular outcomes than noise sensitivity [77]. Weather sensitivity is only moderately correlated with noise sensitivity (see Table S3 in the Supplementary Material). However, weather sensitivity showed a stronger correlation with headaches than noise sensitivity in the current study (r = 0.35 *vs.* 0.17)—which indicates stronger vegetative lability.

On the smaller sample, which included anthropometric measurements, BMI was an additional strong predictor—replicating the role of lower BMI values in the noise-hypotension relationship we have observed in the previous analysis [41]. This distinct body habitus (low body mass, especially in young females) is a well known predictor of hypotension in clinical practice [2]. It has been identified as the most important clinical predictor of hypotensive events in a study using ambulatory blood pressure monitoring [78] and as predictor of low blood pressure in field studies [79]. Furthermore, body fat has been shown to influence autonomic regulation and to change sympathovagal balance. Higher body fat is known to be associated with sympathetic activation [80,81,82,83]. To the contrary, weight loss reduced sympathetic activity [84,85] and was significantly related to an increase in cardiac parasympathetic activity and to lower blood pressure [86]. A group with low BMI showed a significant decrease in the low-to-high frequency (LF/HF) ratio in an experiment using lunch as exposure, indicating parasympathetic system dominance in this group of young female students in contrast to more sympathetic reactions in the high BMI group [87].

Although we still observed a higher prevalence of reported hypotension among young women compared with men, the noise × age interaction in the model could not be replicated. However, age did significantly moderate the relationship with sex (in the models with reported hypotension). As the current study used an extended age range (until 75 years), the relationship in the current model may have been affected by this change of the age range.

Notably, although age and sex were also significant predictors of hypotension medication use (not available in the earlier survey), the age×sex interaction was not significant in the medication models. The observed selection factors involved in the prescription of anti-hypotensive medication is a reasonable explanation for the non-replication of the age × sex interaction with the medication outcome. The age-sex distributions for the reported hypotension and hypotension medication did show quite different shapes (see Figures S2 and S3) in the Supplementary Material).

Due to the omission of the von Zerssen symptom scale in favor of the GHQ we could not replicate the interaction regarding sound exposure and exhaustion. The GHQ did not include an equivalent item and none of the subscales we used (somatic and anxiety) made a stronger prediction compared with the standard health status item we applied instead.

The reported prevalence of hypotension was significantly lower in this study. One obvious reason is the larger proportion of older subjects who exhibit lower rates. However, about 70% of those reporting symptoms of hypotension also consumed medication during the past 12 months. More accurate trends in reporting over time may be another reason, because the ever reported hypotension prevalence in this study was in full agreement with the prevalence obtained in the earlier study. 

Again, measured blood pressure was not a significant outcome in any of the models. One of the main reasons may be that a large fraction of people reporting hypotension episodes take medication (~70%). Furthermore, the number of repeated measurements was probably too small to single out persons suffering from hypotension by blood pressure recordings only. Whether tests for orthostatic hypotension and orthostatic dizziness would help is not sure [88]. While the population prevalence is similar to the one we observed in our population survey the age distribution is quite different [89,90].

Acute air pollution exposure is known to trigger autonomic nervous system imbalance, however the best described direct and indirect pathways mostly lead to up-regulation of the sympathetic nervous system with consecutive elevation in blood pressure [91,92,93]. Nevertheless, we tested air pollution and the potential interaction with noise in the reported models. However, no single or combined effect could be observed and the terms were removed from the final models without any change in the sound estimates.

This intensive (door to door) survey has implemented several improvements in study design, exposure and questionnaire assessment over the previous study. The sample design was not oriented towards municipal boundaries but built around 31 noise measurement points (See Figure S1, Supplementary Material). From 500 m GIS-circles—with the measurement site at the center—persons were sampled at random in a two-step procedure (with replacement) from four noise exposure strata (35–44, 45–54, 55–64, >64 Leq, dBA). This procedure reduces the inaccuracy of the noise engineering propagation models at larger distances.

Another gain from this improved sampling design was a high and balanced number of participants in the central sound level range between 45 and 65 dBA (see Table S4 in the Supplementary Material), where the non-linearity of the observed association between sound and hypotension arises. Furthermore, sound exposure could be differentiated by sources and air pollution assignments were available.

Most of the questionnaire items were exactly the same. Reported hypotension medication was added as second health outcome. The improved sleep scale did underline the importance of poor sleep in this study. The related symptoms (exhaustion, fatigue) included in the earlier analysis may have reduced the predictive power from the reported sleep disturbance. Eventually, the replication of the main results in the smaller sample is reassuring. It can be seen as a kind of additional split sample validation. Moreover, both samples were nearly identical concerning the socio-demographic and health characteristics (compare Table 1 and Tables S1and S5 in the Supplementary Material).

Critical limitations remain obviously in this study. The cross-sectional design prevents a causal interpretation. The sample size is only moderate and the wide confidence intervals show that the models were at its limits in terms of variables related to the number of observations. In any case, the sample size was not large enough to detect potential noise effects based on four casual blood pressure recordings only. Future studies should clarify whether clinical tests for orthostatic hypotension and orthostatic dizziness are helpful to reduce misclassification. Sound exposure assessment should include also the noise from smaller roads. Unfortunately, the GHQ, as used in this study, did provide less detailed information than the von Zerssen symptom scale. The shorter time frame (“last weeks”) of reporting in the GHQ may be one possible reason.

As often, when new relationships are addressed in research, the detailed autonomous mechanisms by which noise exerts its effect on hypotension are not yet well understood. There are, however, several pieces of evidence which are in agreement with the findings of this and the earlier study. The only sound related evidence comes from an experimental study. They found fastest habituation of electrodermal responses in hypotensives after presentation of 16 tones (1000 Hz, 90 dB) compared with borderline hypertensives and normotensives [94]. Growing recent evidence supports the idea of a general cardiovascular down-regulation in essential hypotension [95,96,97,98] and a sympathetic withdrawal has been proposed as underlying this condition by some authors [6,94,99]. An increased baroreflex sensitivity is another mechanism discussed by [98] who demonstrated increased sensitivity of such neural feedback mechanism in chronic hypotensives associated with lower increases of BP and stroke volume under stress.

## 5. Conclusions

The replication of an association between traffic noise, dominated by nightly rail exposure, and hypotension adds credence to the earlier reported results. The current analysis suggest both a partially moderated but also a possible direct non-linear association of total and rail noise with the predicted proportion of hypotension or hypotension medication. Other important cofactors (sex, age, BMI, health) and moderators (weather sensitivity, adjacent main roads and associated annoyance) need to be considered as indispensible part of the observed relationship. Given the prevalence of reported hypotension (14%) and reported medication use (10%) the public health relevance of potential effects of noise is undoubtedly given. Larger studies and replication in different regions and populations are needed to verify the associations and the vulnerability and response characteristics observed in this study.

## Supplementary Files

Supplementary File 1
