# Supplementary material for: Hypotension and Environmental Noise: A Replication Study
Source: Int J Environ Res Public Health. 2014 Aug 26;11(9):8661–88. doi: 10.3390/ijerph110908661 (PMC4198985; doi:10.3390/ijerph110908661)
Supplement: Supplementary File 1 [file ijerph-11-08661-s001.pdf]

Supplementary Material

Hypotension and Environmental Noise: A Replication Study

Figure S1. The sampling procedure around 31 noise measurement points from the three areas of the lower Inn valley, Austria.

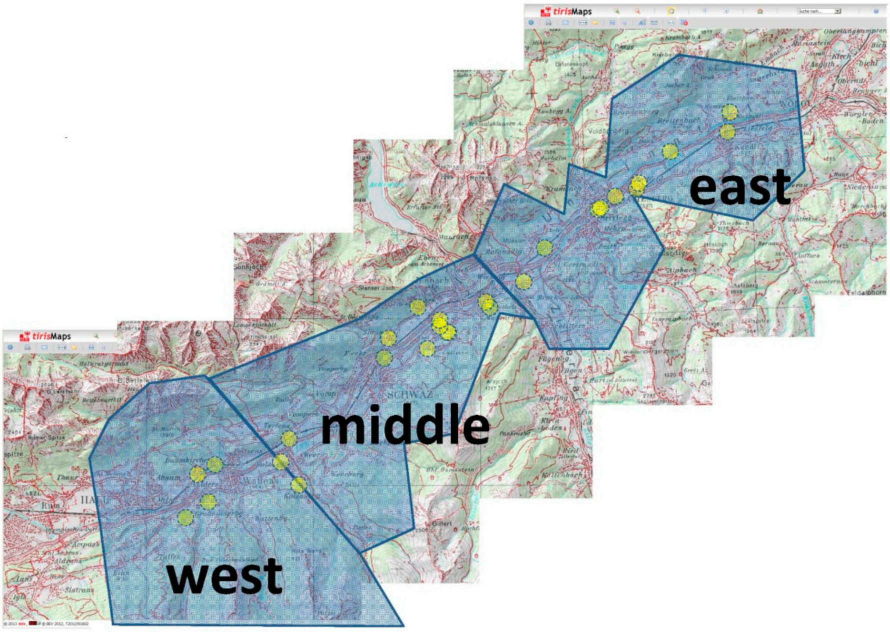

Figure S2. Age-sex distribution of reported hypotension in the reduced sample (**a**) and the full sample (**b**).

(**a**)

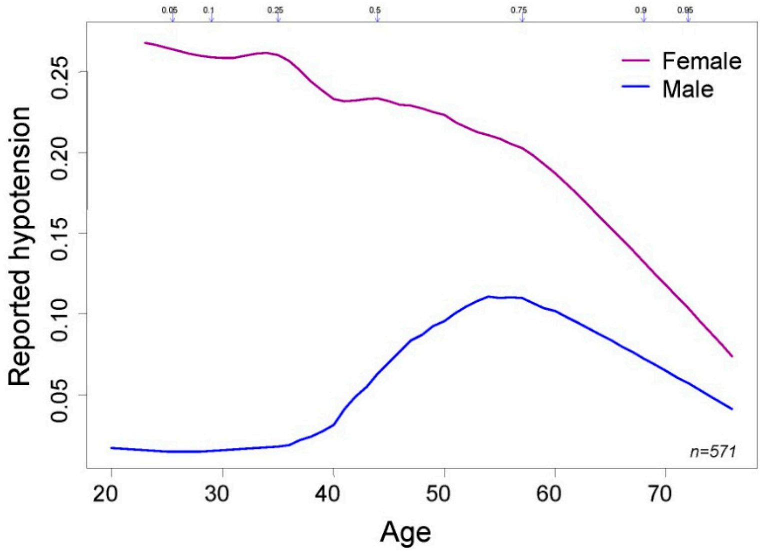

Figure S2. *Cont.*

(**b**)

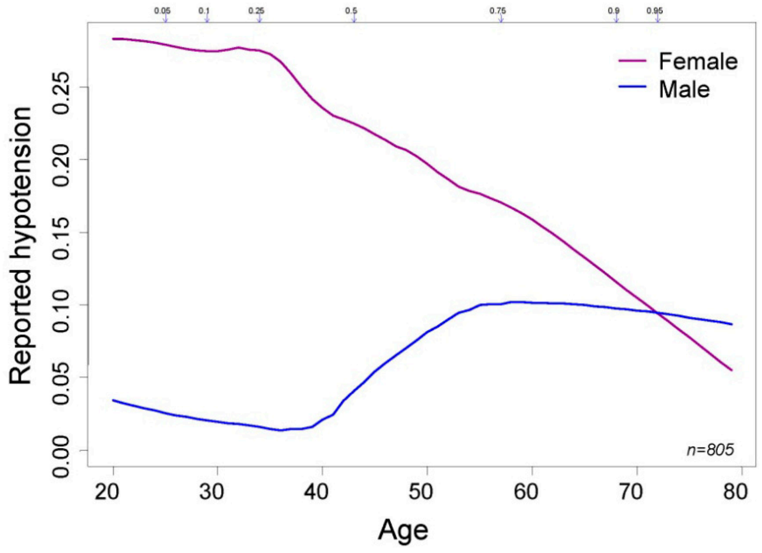

Figure S3. Age-sex distribution of reported hypotension in the reduced sample (**a**) and the full sample (**b**).

(**a**)

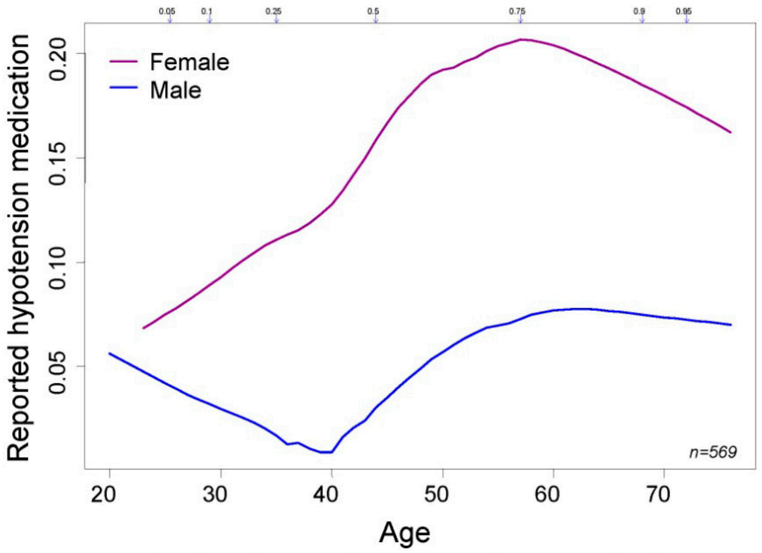

(**b**)

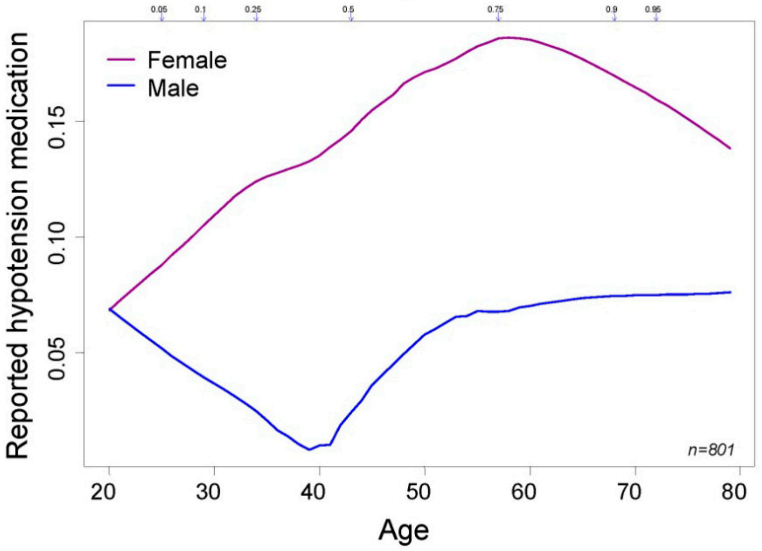

Table S1. Description of the relationships between main study variables and the two health outcomes (reduced sample).

Categorical Variables	Reported Hypotension: No	Reported Hypotension: Yes	Chi-Square-Statistic	Hypotension Medication: No	Hypotension Medication: Yes	Chi-Square-Statistic
	n (%)	n (%)	*p* value	n (%)	n (%)	*p* value
Total	485 (85)	87 (15)		510 (89)	60 (11)	
Gender			<0.001			<0.001
Female	256 (52.8)	73 (83.9)		280 (54.9)	49 (81.7)	
Male	229 (47.2)	14 (16.1)		230 (45.1)	11 (18.3)	
Health status			0.002			<0.001
Very good/good	275 (56.7)	33 (37.9)		296 (58)	12 (20)	
Less than good	210 (43.3)	54 (62.1)		214 (42)	48 (80)	
Educational level			0.335			0.107
Basic	137 (28.5)	22 (25.3)		140 (27.7)	19 (32.2)	
Skilled labor	163 (34)	34 (39.1)		174 (34.4)	21 (35.6)	
Vocational	100 (20.8)	22 (25.3)		106 (20.9)	16 (27.1)	
A-level	80 (16.7)	9 (10.3)		86 (17)	3 (5.1)	
Area of valley			0.742			0.88
East	154 (31.8)	24 (27.6)		160 (31.4)	18 (30)	
Middle	216 (44.5)	41 (47.1)		229 (44.9)	26 (43.3)	
West	115 (23.7)	22 (25.3)		121 (23.7)	16 (26.7)	
Family history of hypertension			0.006			0.294
Yes	157 (32.4)	42 (48.3)		173 (33.9)	25 (41.7)	
No	327 (67.6)	45 (51.7)		337 (66.1)	35 (58.3)	
Antihypertensive treatment			<0.001			0.006
No	388 (80.3)	85 (98.8)		416 (81.6)	57 (96.6)	
Yes	95 (19.7)	1 (1.2)		94 (18.4)	2 (3.4)	

Table S1. *Cont.*

**Continuous Variables**	**Reported Hypotension: No Median (IQR)**	**Reported Hypotension: Yes Median (IQR)**	***p* Value Ranksum Test**	**Hypotension Medication: No Median(IQR)**	**Hypotension Medication: Yes Median(IQR)**	***p* Value Ranksum Test**
Age			0.294			0.02
median (IQR)	44 (35, 58)	42 (34.5, 54)		43 (34, 57)	52 (40, 60)	
Body mass index			<0.001			0.002
median (IQR)	26 (23.1, 28.8)	23.5 (21.5, 26.4)		25.9 (22.9, 28.7)	24.3 (21.7, 26.6)	
Total sound level: dBA, Ldn+			0.191			0.87
median (IQR)	58.2 (54.7, 61.7)	58.7 (55.2, 64.8)		58.3 (54.9, 61.9)	57.7 (53.8, 64.3)	
Rail sound level: dBA, Ldn+			0.292			0.915
median (IQR)	55 (52, 60.1)	55.2 (52.3, 63.8)		55.2 (52.2, 60.2)	54 (51.7, 63.9)	
Highway sound level: dBA, Ldn+			0.546			0.937
median (IQR)	54.2 (50.2, 56.5)	54.2 (51, 57.2)		54.2 (50.4, 56.6)	54.2 (49.3, 57.4)	
Distance to main road			0.024			0.404
median (IQR)	472.2 (200.2, 1202.1)	306.1 (160.5, 1129.6)		438.2 (185.1, 1193)	380 (171.4, 1136.7)	
Annoyance by local road			0.318			0.004
median (IQR)	4 (2, 6)	5 (2, 6)		4 (1.8, 6)	5 (3, 7.2)	
NO2: annual average, µg/m³			0.483			0.617
median (IQR)	34 (32.2, 36.1)	34.4 (32.4, 36.4)		34 (32.2, 36.2)	33.9 (32.1, 35.8)	
Noise sensitivity *			0.022			0.019
median (IQR)	5 (2, 8)	6 (3, 8)		5 (2, 8)	6.5 (3, 9)	
Weather sensitivity *			<0.001			<0.001
median (IQR)	3 (1, 5)	5 (3.2, 8)		3 (1, 5)	6 (4, 8)	
GHQ score *			<0.001			<0.001
median (IQR)	21 (18, 26)	24 (20, 29.8)		21 (18, 26)	25 (21, 30.8)	
Sleep score *			0.002			
median (IQR)	6 (3, 10)	9 (4, 13)		6 (3, 10)	10 (6, 14.5)	
Systolic BP: 2nd reading			<0.001			<0.001
median (IQR)	136 (123, 150)	121 (110, 132)		135 (121, 150)	125.5 (111.5, 136)	
Diastolic BP: 2nd reading			<0.001			<0.001
median (IQR)	89 (80.2, 96)	83 (73.5, 88.5)		88.5 (80, 96)	84.5 (75, 90)	
Systolic BP: 4th reading			<0.001			<0.001
median (IQR)	134 (120, 146)	116 (110, 128)		132 (118, 146)	122 (112, 135.2)	
Diastolic BP: 4th reading			<0.001			0.061
median (IQR)	86 (79, 94)	78 (72, 85.5)		85 (78, 93.2)	82.5 (76.8, 88.2)	

* The higher the worse; + Ldn: day-night adjusted sound level in decibel.

Table S2. Sound levels of highway and railway by day and night in both samples.

Sound Source	Sound Level, dBA—Reduced Sample			Day-Night Difference	Sound Level, dBA—Full Sample			Day-Night Difference
	5th Percentile	Median	95th Percentile		5th Percentile	Median	95th Percentile	
**Highway**								
Day	40.4	52.9	59.8	6.9	41.4	52.5	59.6	6.9
Night	33.5	46.0	52.9		34.5	45.6	52.7	
**Railway**								
Day	37.7	46.6	63.8	−2.8	38.0	45.8	63.3	−2.8
Night	40.5	49.4	66.6		40.8	48.6	66.1	

Table S3. Correlation between different sensitivities in the full sample (N = 807).

Sensitivity Type	Weather Sensitivity	Noise Sensitivity	Air Pollution Sensitivity	Vibration Sensitivity
Weather sensitivity	1.00	0.30	0.37	0.18
Noise sensitivity	0.30	1.00	0.64	0.66
Air pollution sensitivity	0.37	0.64	1.00	0.57
Vibration sensitivity	0.18	0.66	0.57	1.00

Table S4. Sex, reported hypotension, hypotension medication by sound level of sources.

Sound Level, dBA, Ldn	Sex		Hypotension Reported		Hypotension Medication	
	Female	Male	No	Yes	No	Yes
**Highway**						
<45	42	36	68	10	68	10
45–54	224	196	363	57	378	39
55–64	149	130	238	41	252	26
65+	0	3	3	0	3	0
Sum	415	365	672	108	701	75
**Railway**						
<45	8	6	12	2	10	3
45–54	214	195	354	55	368	39
55–64	139	127	235	31	247	18
65+	54	37	71	20	76	15
Sum	415	365	672	108	701	75

Table S4. *Cont.*

Sound Level, dBA, Ldn	Sex		Hypotension Reported		Hypotension Medication	
	Female	Male	No	Yes	No	Yes
**Overall sound level**						
<45	4	4	8	0	7	1
45–54	118	103	192	29	197	22
55–64	235	216	393	58	412	37
65+	58	42	79	21	85	15
Sum	415	365	672	108	701	75

Table S5. Sociodemographic characteristics in both samples and census.

Indicator	Overall Comparison of Samples		
	Full Sample	Reduced Sample+	Census
Male sex	46.8	42.5	48.0
Female sex	53.2	57.5	52.0
Higher education: 12+ years	19.0	16.0	20.5
Basic education: 9 years	25.0	28.0	N.A.
Noise sensitivity: high	27.0	29.0	N.A.
Weather sensitivity: high	20.0	21.0	N.A.
Health status less than good	46.0	46.0	N.A.
Health status very good	15.0	16.0	N.A.
Hypotension ever	28.9	29.5	N.A.
Hypotension reported *	14.1	15.2	N.A.
Hypotension medication *	9.8	10.5	N.A.
Hypertension medication *	15.5	16.1	N.A.

+ with anthropometric measurements; * past 12 month.

© 2014 by the authors; licensee MDPI, Basel, Switzerland. This article is an open access article distributed under the terms and conditions of the Creative Commons Attribution license (http://creativecommons.org/licenses/by/3.0/).
